# Loot

**Published:** 1875-08

**Authors:** 


### Loot.

### A NOVEL TREATMENT OF OBSTINATE VOMITING IN PREGNANCY.

By Edward Copeman, M.D., P.R.C.P., Senior Physician to the Norfolk and Norwich Hospital, President of the British Medical Association.

On June 9, 1874, I was summoned to a lady, thirty-five years of age or thereabouts, to consult with two other practitioners already in attendance. She was about six months gone in pregnancy, and was so reduced by almost incessant vomiting that great fears were entertained as to her safety. I noticed there was slight uterine action accompanying the sickness, and, on examination, I found the os uteri partially dilated so as readily to admit the finger. I thought it right, under the emergency, to advise bringing on labor without delay; the gentlemen present, however, expressed no little apprehension as to whether or not she would have strength to undergo the effort of parturition on account of the very depressed and exhausted state of her system. They nevertheless concurred in the advisability of the course I recommended, and asked me to perform the operation. I at once dilated the os uteri as much as I could with the finger, and could feel the membranes and the head of the child. I tried to rupture the membranes with a telescopic female catheter (the only instrument at hand), but they were so flaccid and the head offered so little resistance, the catheter shortening itself also on my making pressure, that I could not succeed; and, thinking it wise to wait awhile before resorting to any other expedient, we retired to another room for further consultation. In about an hour we saw the patient again, and were surprised to find that a longer period had elapsed without sickness than before; and we again waited, in the hope that she might be able to take a little nourishment, and so be prepared to undergo any further proceedings. We waited another hour, and then another, but there was no return of vomiting; and we spent the rest of the night in watching, during the whole of which time she was improving, and we determined to let well alone. I left her early in the morning, and had a favorable account of her a few days afterwards. There was no return of sickness; she went on to the full period of pregnancy, was then delivered of a healthy child, and made a good recovery.

This case made a strong impression on my mind; and I wondered whether the relief to the vomiting, so urgent and threatening to her life, could have been effected by my having dilated the os uteri, and thus removed any undue tension that might be producing sympathetic irritation. It was not very long before I was called some distance into the country to consult about another case of vomiting during pregnancy of great urgency, occurring about the second month. The surgeon in attendance had adopted the best acknowledged medical treatment, and had arrived at the conclusion that artificial delivery would be necessary to save her life. With the full recollection of the former case, I examined the uterus, and found some degree of anteversion and the os patent enough to admit the end of my finger. I forthwith dilated it as much as I could, passing my finger all round, removing all puckering and making a smooth edge. She vomited only once slightly after this proceeding, and we left her with the understanding that, if the sickness continued, I should be summoned again in a few days to bring on abortion. This summons never came; but in about a fortnight I had a letter from the husband, stating that his wife began to get better an hour or two after I left, and that the sickness had entirely ceased. I have heard several times since that the patient is going on remarkably well, and I believe she expects to be confined some time this month.

A third opportunity has since offered itself for a trial of this novel (as far as I know) treatment. On the 6th of the month (April, 1875,) I saw, in consultation with a very intelligent country practitioner, a lady in delicate health just entering the eighth month of pregnancy. She was the mother of nine or ten children, and her life was valuable. Generally during early pregnancy, and sometimes for several months together, she had been troubled with vomiting ; but during the last three weeks, the sickness had been almost incessant ; she could keep nothing down, and was in a very feeble and emaciated condition. She had, moreover, a considerable amount of albumen and some pus in the urine, a few casts also ; and fears were entertained of there being extensive kidney disease. There was, however, no dropsy, and our opinion was somewhat modified by the knowledge that the urine does often, during pregnancy in the latter months, contain a great deal of albumen. The patient was so ill that she would willingly have consented to artificial delivery, if really necessary. I examined the uterus, and, as in the other cases, found it patent, puckered, and dilatable, and I dilated it as much as I could with my finger, in the hope that the sickness might cease after such a proceeding. I should say that the usual remedies had been carefully employed without producing the desired effect. A few days after my visit, her husband called upon me to say that his wife had no return of sickness after I left, and was now able to take food without inconvenience, although he still thought her very weak and ill, and feared she would not recover.

On the 23rd, I received a very satisfactory letter from the surgeon in attendance, to the following effect: "There was never any urgent sickness after you dilated the os uteri, and the last week Mrs. —— has frequently taken with relish and no inconvenience solid food, such as boiled mutton, with asparagus, and drunk home-brewed beer. This morning she was going on quite well ; she was not even faint or at all exhausted after her labor. I am very glad I called you in, for I now know how to proceed with cases of sickness in pregnancy. Should I meet with any more such patients, I will either ask you kindly to meet me again, or report them to you."—*British Med. Journal.*

### PREVENTIVE MEASURES IN SYPHILIS.

Mr. Acton recently read a paper before the Royal Medical and Chirurgical Society of London, from which we make the following extracts:—

His paper commenced by stating that when he returned to England, after the completion of his studies in Paris, he was greatly struck with the severity and number of cases of syphilis in London, as compared with Paris, and as a consequence of this he brought the subject before the notice of the Society in 1846, and again in 1860, showing that the Belgian and French troops were much less attacked by venereal affections than the English. In 1873, he found that in districts in England where the troops were not what he called protected from the women, primary syphilis still existed in the proportion of 123 per 1,000 men annually. He maintained that syphilis could be prevented and stamped out by providing ready means of ablution, and destroying the local form of contagion, and warning male patients not to infect other persons. The institution of hospitals, whether free or otherwise, was one remedy, for treatment of prostitutes as out-patients was quite inadequate. They should be segregated as soon as diseased, and not allowed to leave hospital until they are quite cured. By doing this, as at Hong Kong and Dartmouth, the disease had been reduced to a minimum. In his visit to Brussels, in 1874, Mr. Acton had visited the Military Hospital, where he found only three cases of syphilis among the private soldiers, and two among the non-commissioned officers, out of a body of 3,500 troops. There were only nine women confined to hospital for venereal disease, showing that in Brussels the police inspection had nearly stamped out the disease. In Paris he visited the military hospitals, and could only discover six cases of primary disease, and eight of secondary syphilis, among 3,841 men forming the garrison of Paris. Disease among the females was very slight also, and Mr. Acton attributed this decrease to the police regulations. He gave a table showing that in the St. Lazare Hospital he only found 23 cases of primary disease among 202 patients in this prison, which is under the police surveillance. With respect to England, Mr. Acton said that Parisian medical men alleged that British travelers, like sailors, were the cause of much of the disease in Paris, and that the disease would ere now have been stamped out had it not been that England and other similar countries went on continually introducing fresh cases into Paris. In London he found at the hospital of the Foot Guards 24 cases of primary disease among 408 single soldiers in the second battalion of the Coldstream Guards quartered in London. In the first battalion of the Scots Fusilier Guards he found 25 cases of severe forms of syphilis among 505 unmarried men. He handed in a table extending over a year, which showed that one-fifth of the whole troops quartered in London in 1874 were affected with primary sores, which would have incapacitated the men from duty for a period of six weeks on an average. Perhaps 164 of these men would have secondary disease, requiring mercury, which would further incapacitate them from duty for a period of two months or so, and this would debilitate them greatly. Comparing the syphilic affections of the Foot Guards with those among the troops quartered in Paris, he showed that 500 troops in London had more disease than 3,841 quartered in Paris. Mr. Acton considered that one-half the prostitututes in London were diseased ; whereas of those in the districts under the Contagious Diseases Acts only about eight per cent. were found affected at periodical examinations. It appeared that at Woolwich, during 1871-2-3, only 1,085 cases of primary sores were treated in hospital, out of a garrison of 18,250 men, or only one man was infected in seventeen soldiers, instead of one in six, as in London. He therefore, in conclusion, looked upon the advantages of supervision of prostitutes as no longer a problem, but as an undoubted fact.—*M. & S. Reporter.*

---

### VOCAL FREMITUS IN PLEURISY AND PNEUMONIA, AND THE USE OF THE HYPODERMIC SYRINGE IN THEIR DIAGNOSIS.

By E. G. JANEWAY, M.D., New York.

The value of vocal fremitus as a physical sign in the diagnosis of pleurisy, with effusion from pneumonia, is, it seems to me, not infrequently rated too highly. Some, in fact, appear to consider it an absolute means of distinguishing the one from the other condition. My reasons for not esteeming it as much as those who hold this opinion, are based upon the observation of cases in which such a belief would have been or proved to be a mistake. Let me first give a brief record of two cases of pleurisy with effusion, in which vocal fremitus was present over the affected portion. The first of these was a male patient in Ward 8 of Bellevue Hospital last summer (Simms), age 33.

He was taken sick two days before admission with rigors and a pain in the left side. When admitted the physical signs of interest were: Flatness on percussion on the right side of the chest below the angle of the scapula, increase of vocal fremitus over the affected portion, feeble and distant respiratory sounds, ægophonic vocal resonance.

June 3d, two days later, distinct vocal fremitus was present over the affected portion, and a hypodermic syringe withdrew clear serum from the pleural sac at this part.

Aspiration of the affected side was performed several times, with only partial relief, and in consequence of obstruction of the needle, or failure of the apparatus, on two occasions but little fluid was obtained. After the second aspiration air was detected in the pleural cavity above the level of the remaining fluid, but below this vocal fremitus persisted. Later sero-pus and pus formed in the cavity, and it was decided to make an opening in the pleura, and allow the fluid to drain off. The reasons for the operation were the great dyspnœa, the existence of pus in the sac, the failure of aspiration to afford relief which was more than temporary, the increasing weakness of patient, and finally paroxysms, during which the patient would pass almost into a state of collapse. The day following the incision vocal fremitus disappeared from the affected portion. On the 12th of November the patient was discharged.

The second patient was a male, fifty years of age, admitted to Bellevue Hospital on the 17th of November, 1874. His illness had commenced one month before, with symptoms which pointed to the existence of pneumonia on the left side. When admitted he was thought to have a chronic pneumonia, or pleurisy with pneumonia of left lower lobe, extending into upper. Of this man I made a very careful examination about a week before his death. The following physical signs were then present: Flatness on percussion existed over the lower lobe of the left lung, except near its most anterior portion; here there was some resonance.

Bronchial voice, whisper, and respiratory murmur were heard over this part more distinct above, more distant and feebler below.

Measurement showed the two sides to be of equal size. The heart was not displaced, and vocal fremitus existed over the affected portion, and was increased as compared with the opposite side.

There was no change of the level of flatness on percussion, and this line of flatness corresponded very closely to the interlobar division. The character of the bronchial breathing and voice made me suspect fluid, and so at my request the House Physician, Dr. Chapin, introduced the needle of a hypodermic syringe in the pleural sac, but failed to obtain any fluid. The hypodermic syringe seemed to be good, as tested by drawing up water from a tumbler. I then proposed bringing an aspirator on the succeeding day, for more thorough exploration, but the patient passed into a typhoid state, seeming to indicate the existence of meningitis as a complication. A post-mortem examination revealed the following: Dilatation of the ventricles of the brain, with a granular condition of the ependyma, and empyema of the left side, correspond ing to the site of the left lower lobe, and shut in by adhesions of the upper lobe. A small portion of the anterior part of the lower lobe was adherent to chest, near heart, and the rest was carnified and pressed upwards, inwards and backwards.

The heart was not displaced. The lower part of the upper lobe of the left lung was hepatized. There was no adhesion of the lung over the space where I had perceived vocal fremitus ; nor was any band-like adhesion, such as sometimes occurs, present.

These two cases, which have been observed within a period of eight months, are sufficient to show that vocal fremitus may be present over fluid in the pleural sac, and may even be exaggerated. The disappearance of the vocal fremitus, in the one case after the removal of the fluid, and the results of the post-mortem examination in the other, show that the vocal fremitus was conducted through fluid to the chest wall.

In addition to these cases, I have observed two others during the same period, in one of which a slight vocal fremitus existed over fluid in the left pleural sac. This was enough to cause one physician to doubt the correctness of the diagnosis, though the left side was two inches larger than the right, and the heart displaced. He, however, confessed when a hypodermic syringe drew fluid from this portion, and an aspirator removed sixty ounces of serum.

In the other case the vocal fremitus was increased, but of this I will make no use, as the patient escaped from my observation, before I was able, satisfactorily, to determine the conditions present.

Again, cases present themselves in which the vocal fremitus is exceedingly feeble or absent on the healthy side, and then the absence of this sign has no especial value on the diseased side.

Such a case I have lately seen in a young man with rheumatic pericarditis and a small effusion in the left pleural sac. In this case vocal fremitus is absent at corresponding points on the two sides, but above the level of the fluid, for a short distance, it is considerably increased on the left or affected side: while at a corresponding point on the right side it is only feebly perceptible. Bronchial character of the expiratory sound is audible over the fluid, and broncho-ægophony exists near its upper limit. I can very readily imagine that an incomplete examination might lead to the supposition of pneumonia. While I have been writing this article the fluid has accumulated somewhat on the right side also, but the vocal fremitus remains as at first. On the other hand cases have occurred in which pneumonia has been mistaken for pleurisy, with effusion, by very good observers.

Some years ago I made the post-mortem examination in a case of supposed pleuritic effusion. The autopsy revealed a small aneurism resting on the left main bronchus, and this completely occluded by a firm thrombus which had formed by the escape of blood from the aneurism into it by means of a small opening. The left lung was throughout in a state of gray hepatization, and its bronchi were filled with purulent fluid. Those who had seen this case, during life, stated that all the signs of pleuritic effusion had existed, absence of vocal fremitus, of respiratory murmur, of vocal resonance, with the existence of flatness on percussion. If any suspicion of the real condition of affairs had been entertained it seems probable that absence of displacement of the heart, and of increase of the measurement of the affected side might have corrected the diagnosis, considering the extent of disease. Since that time I have seen a second nearly identical case.

Again it sometimes happens that with ordinary pneumonia producing complete solidification of a lobe, considerable diminution or absence of the vocal fremitus may occur.

Allow me, in closing this short article, which is written in order to draw attention to the need of caution in the interpretation of physical signs, to urge the importance of attending to each physical sign in doubtful cases of these two diseases, and also of examining the symptoms presented by the patients, and the course which the disease takes. In doubtful cases, where, if fluid should exist, the line of treatment would be decidedly different, I think it wise to make use of an exploratory puncture. This may be done by means of a small needle attached to the aspirator, or, as this not infrequently alarms patients, at Bellevue Hospital a hypodermic syringe is often employed for this purpose. Considerable difference exists in the suction power of these instruments, and I have seen one introduced in a chest and fail to obtain fluid when another immediately afterwards succeeded. The safest test is not that of seeing whether water can be drawn through the needle into the barrel of the syringe ; but, placing a finger over the nozzle, withdraw the piston and retain it in this position for a few seconds,. and then note if the atmospheric pressure will force the piston back to its original position. With one, which answers perfectly to this test, I have succeeded in drawing pus from abscesses ; and lately, in order to give it a fair test and also to relieve a patient, I employed it to remove thick gelatinous fluid from a ranula. This fluid was a little more viscid than the mucus of cervix uteri. If honest doubt existed after its use it would be wise to make use of an instrument with greater suction force.

Summary of the conditions of vocal fremitus in pleurisy and pneumonia :

1st. Local fremitus is usually absent over fluid in the pleural sac.

2nd. When present (*a*) it is sometimes due to adhesions of the lung to the chest wall below the level of the liquid.

*b.* Again, it is sometimes conducted through the fluid, and then it may be either feebler or more intense than on the opposite side.

*c.* In some cases vocal fremitus does not exist or is very feeble at the lower part of the chest on the sound side ; under these circumstances its absence on the diseased side loses some of its relative value.

3rd. Vocal fremitus is usually increased over pneumomia of the lower lobe.

4th. It may, however, be very feeble or absent.

*a.* This may be due to obstruction of the bronchus by compression, or by the accumulation of some material in it, as, in one of the cases reported, coagulated blood.

*b.* Sometimes it seems due to the presence of a considerable amount of exudation in the pleural sac.

*c.* The amount of solidification is supposed at times to be the cause.

5th. Vocal fremitus is sometimes absent on the sound side ; under such circumstances the existence of considerable vocal fremitus on the diseased would point strongly towards, though not be decisive of, pneumonia, as the morbid condition present.

My friend and colleague, Prof. Flint, is also in the habit of drawing attention to the greater value of absence of vocal fremitus on the right side as a sign of pleuritic effusion, and of its exaggeration on the left side as a sign of pneumonia, in view of the normal difference of vocal fremitus on the two sides.

It will be obvious, that in this article I have considered that the more important physical signs, percussion and auscultation, have pointed out the site of the disease, but have not been able to establish the diagnosis.—*N. Y. Medical Record.*

---

### *Illinois State Medical Society.*

SECRETARY'S OFFICE,

296 W. MONROE ST., CHICAGO, May 25 1875.

*Dear Doctor*—At the 25th Annual Session of the Illinois State Medical Society, held in the city of Jacksonville on the 18th, 19th and 20th days of May, 1875, the following officers were elected, and committees and delegates appointed for the ensuing year; also, the appended resolution was adopted, and the Secretary instructed to furnish a copy of said resolution to each and every member of the Society.

OFFICERS :

*President*—THOMAS D. WASHBURN, M.D., Hillsboro.
*First Vice-President*—J. L. WHITE, M.D., Bloomington.
*Second Vice-President*—JOHN WRIGHT, M.D., Clinton.
*Treasurer*—JOHN H. HOLLISTER, M.D., Chicago.
*Permanent Secretary*—T. DAVIS FITCH, M.D., Chicago.
*Assistant Secretary*—C. B. JOHNSON, M.D., Tolono.

STANDING COMMITTEES:

Board of Censors—E. Wenger, Gilman, Chairman; E. Ingals, Chicago; E. L. Herriott, Grafton.

Committee of Arrangements—S. H. Birney, Urbana, Chairman; A. T. Dana; J. T. Pearman, Champaign.

Practical Medicine—Wm. Massey, Grandview, Chairman; D. L. Jewitt, Watseka; J. W. Fink, Hillsboro.

Committee on Surgery—Moses Gunn, Chicago, Chairman; A. C. Rankin, Loda; John G. Hays, Paris.

Committee on Obstetrics—M. W. Walton, Ridott, Chairman ; C. Goodbrake, Clinton; D. E. Foote, Belvidere.

Drugs and Medicines—John H. Etheridge, Chicago, Chairman ; T. J. Pitner, Jacksonville ; H. H. Hood, Litchfield.

Necrology—G. W. Albin, Neoga, Chairman ; Wm. Chambers, Charleston ; J. N. Niglas, Peoria.

Ophthalmology—E. L. Holmes, Chicago.

Otology—F. C. Hotz, Chicago.

SPECIAL COMMITTEES:

Diseases of Children—J. S. Williams, Otterville.

Medical Jurisprudence—M. A. McClellan, Knoxville.

Dermatology—J. Nevins Hyde, Chicago.

Progress in Physiology—Sarah Hackett Stevenson, Chicago.

Idiocy—C. T. Wilbur, Jacksonville.

Neurology—Walter Hay, Chicago.

Gynæcology—A. Reeves Jackson, Chicago.

Hygiene—Jehu Little, Leroy.

Diagnosis and Extirpation of Tumors—W. L. Goodell, Effingham.

Empiricism—J. F. Todd, Galva.

Prudence in the Administration of Therapeutic Agents—E. Ingals, Chicago.

Uterine Displacements—T. Davis Fitch, Chicago.

Staphyloraphy—David Prince, Jacksonville.

Electro-Therapeutics—Plym. S. Hayes, Chicago.

The Treasurer and Secretaries constitute the Committee on Publication.

DELEGATES TO THE AMERICAN MEDICAL ASSOCIATION.

S. C. Plummer, Rock Island; Walter Hay, Chicago; J. F. Todd, Galva; J. B. Rood, Lemont ; F. B. Haller, Vandalia; D. E. Foote, Belvidere ; J. W. Freer, Chicago ; J. H. Hollister, Chicago ; T. P. Peirce, Lemont; Moses Gunn, Chicago ; N. S. Reed, Chandlerville ; H. B. Buck, Springfield ; Thomas J. Whitten, Irving ; Wm. L. Goodell, Effingham ; David Prince, Jacksonville ; G. Wheeler Jones, Danville ; J. Adams Allen, Chicago ; E. P. Cooke, Mendota ; Dr. Darrah, Urbana ; M. W. Walton, Ridott; M. Shepard, Adams Co.; C. Goodbrake, Clinton; J. O. Hamilton, Jerseyville ; Lester Curtis, Chicago ; T. Davis Fitch, Chicago ; E. R. Williard, Wilmington ; William A. Haskell, Edwardsville; Robert Boal, Peoria ; C. T. Wilbur, Jacksonville ; J. M. Steele, Grandview ; L. Clark, Rockford ; O. Everett, Dixon ; T. D. Washburn, Hillsboro ; William A. Elder, Bloomington; C. Paul Simon, Chicago; E. Ingals, Chicago.

DELEGATES TO STATE MEDICAL SOCIETIES.

*Iowa*—Walter Hay, Chicago ; T. Davis Fitch, Chicago.

*Missouri*—F. B. Haller, Vandalia.

*Indiana*—W. W. McMann, Gardner.

*Minnesota*—S. C. Plummer, Rock Island.

*New York*—W. P. Peirce, Lemont.

RESOLUTION.

*Resolved*, That at the next, and all subsequent meetings, all Reports of Committees and other Papers designed for publication in the Transactions of this Society, be presented in a form ready for publication, and placed, when read, at the disposal of the Publishing Committee.

Yours, very truly,

T. DAVIS FITCH,

*Permanent Secretary.*

### *International Congress of Ophthalmology.*

The International Congress of Ophthalmology will meet in New York City, on Tuesday, September 12, 1876, at twelve o'clock noon. The following extracts from the rules of the Congress will give an idea of the general character of the Society, and of the terms of membership :

"1. The object of the International Periodic Congress of Ophthalmology is to promote ophthalmological science, and to serve as a centre to those who cultivate it. It will entertain no discussion foreign to this object.

"2. The number of members is unlimited.

"3. Every member must be either a doctor of medicine, or surgery, or of science, or possess some other equivalent degree, or be distinguished for his scientific knowledge.

"4. Candidates for admission into the Society shall be admitted on presentation of their diploma or of their scientific title, unless ten members demand a ballot.

"5. The sessions of the Society shall take place every fourth year, and be limited to ten days."

"11. The Society gives no diploma. Before the opening of each session a card available for admission to all the meetings, and signed by the President and Secretary, shall be given to each member on payment of his subscription (fixed at $2), and upon signature of his name on the register of those attending the meeting."

Among the members of this Congress, are such men as Arlt and Stellwag, of Vienna ; Giraud-Teulon, Javal, and Wecker, of Paris ; Helmholtz, of Berlin ; William Bowman, George Critchett, R. Liebreich, J. W. Hulke, and Soelberg Wells, of London ; Donders and Snellen, of Utrecht, Holland.

It is hoped that many of them will come to New York in 1876. The committee are making all efforts to secure a large attendance, and one that will leave its mark upon the progress of scientific ophthalmology. The co-operation of the profession of the United States, in securing these objects, is earnestly desired by the undersigned, the Provisional Committee appointed in London, in 1872.

CORNELIUS R. AGNEW, M.D.
HENRY D. NOYES, M.D.
DANIEL B. ST. JOHN ROOSA, M.D.

